# First do no harm - interventions during labor and maternal satisfaction: a descriptive cross-sectional study

**DOI:** 10.1186/s12884-018-2054-0

**Published:** 2018-10-24

**Authors:** Kıymet Yeşilçiçek Çalik, Özlem Karabulutlu, Canan Yavuz

**Affiliations:** 1 Obstetrics and Gynaecology Nursing Department, Karadeniz Technical University, Faculty of HealthScience, University District, Farabi Street, Ortahisar, Trabzon, Turkey; 2 Department of Midwifery, Kafkas University, Faculty of Health Sciences, Kars, Turkey; 3 Midwife, Tekirdağ Community Health Center, Tekirdağ, Turkey

**Keywords:** Interventions during labor, Birth, Maternal satisfaction, Turkey

## Abstract

**Background:**

Interventions can be lifesaving when properly implemented but can also put the lives of both mother and child at risk by disrupting normal physiological childbirth when used indiscriminately without indications. Therefore, this study was performed to investigate the effect of frequent interventions during labor on maternal satisfaction and to provide evidence-based recommendations for labor management decisions.

**Methods:**

The study was performed in descriptive design in a state hospital in Kars, Turkey with 351 pregnant women who were recruited from the delivery ward. The data were collected using three questionnaires: a survey form containing sociodemographic and obstetric characteristics, the Scale for Measuring Maternal Satisfaction in Vaginal Birth, and an intervention observation form.

**Results:**

The average satisfaction scores of the mothers giving birth in our study were found to be low, at 139.59 ± 29.02 (≥150.5 = high satisfaction level, < 150.5 = low satisfaction level). The percentages of the interventions that were carried out were as follows: 80.6%, enema; 22.2%, perineal shaving; 70.7%, induction; 95.4%, continuous EFM; 92.3%, listening to fetal heart sounds; 72.9%, vaginal examination (two-hourly); 31.9%, amniotomy; 31.3%, medication for pain control; 74.9%, intravenous fluids; 80.3%, restricting food/liquid intake; 54.7%, palpation of contractions on the fundus; 35.0%, restriction of movement; 99.1%, vaginal irrigation with chlorhexidine; 85.5%, using a “hands on” method; 68.9%, episiotomy; 74.6%, closed glottis pushing; 43.3%, fundal pressure; 55.3%, delayed umbilical cord clamping; 86.0%, delayed skin-to-skin contact; 60.1%, controlled cord traction; 68.9%, postpartum hemorrhage control; and 27.6%, uterine massage. The satisfaction levels of those who experienced the interventions of induction, EFM, restriction of movement, two-hourly vaginal examinations, intravenous fluid, fundal pressure, episiotomy, palpation of contractions on the fundus, closed glottis pushing, delayed umbilical cord clamping, delayed skin-to-skin contact, fluid/food restriction, and of those who were not provided pharmacological pain control were found to be lower (*p* < 0.05).

**Conclusion:**

Medical interventions carried out at high rates had a negative impact on women’s childbirth experience. Therefore, a proper assessment in the light of medical evidence should be made before deciding that it is absolutely necessary to intervene in the birthing process and the interdisciplinary team should ensure that intrapartum caregivers will “first do no harm.”

## Background

*“A natural birth that takes place of its own accord without interventions of any sort is a complicated process in itself, but also equally fine-tuned and balanced, prone to having its optimal properties eliminated with each intervention. Therefore, the only intervention asked of those supervising childbirth should be to respect this awe-inspiring phenomenon and adhere to medicine’s first fundamental principle which reads ‘Pimum non nocere’* [1]*.”* Indiscriminately resorting to medical intervention where it is not needed violates the principle of respect for medical physiology and the fundamental medical principle of “PRIMUM NON NOCERE.”

Advances in medical technologies have undeniably provided significant benefits in terms of maternal and infant health, especially in high-risk pregnancies and premature births. In recent years in some countries, however, almost all pregnant mothers undergo interventions (enema, perineal shaving, liquid and food intake restrictions, routine IV fluid infusion, continuous EFM (electronic fetal monitoring), routine amniotomy, frequent vaginal examinations, use of vaginal antiseptic agents) without proper assessment of whether it is really needed [2–7]. For example, studies conducted in countries in Latin America, the Caribbean, Canada, Spain, China, South Africa and Turkey indicate that unnecessary medical interventions are common during normal labor [2–7]. It is known that unnecessary interventions undertaken without indication disrupt the natural progression of labor, causing complications to the fetus and the mother (ketosis, dehydration, prolonged labor, interventional delivery, postpartum hemorrhage, hypoglycemia, hyponatremia, cost increase, restriction of options for subsequent births, negative and unhappy childbirth experiences, feelings of failure and guilt, longer hospitalization periods, etc.) [8, 9]. For example, in many hospitals, obstetric interventions such as eating and drinking restrictions, IV fluid infusion, continuous EFM, induction, enema and episiotomy are routinely performed on all women without a specific medical justification [3, 4, 6, 8–11]. In fact, recent evidence-based studies indicate that routine interventions during low-risk births have failed to make births safer for either the mother or the baby, and that some medical practices disrupt the natural course of birth, creating unwanted complications during labor. Furthermore, such interventions tend to cause women to be dissatisfied with the childbirth experience, causing them to seek alternative methods for their next child’s birth [1, 2, 8]. Care must therefore be taken to maximize the use of preventive measures during the normal delivery process to minimize the need for interventions [8]. The World Health Organization (WHO) envisions a world in which all pregnant women and their babies are provided quality care during pregnancy, birth and the postnatal period. In order to reduce maternal and infant morbidity and mortality, every pregnant woman needs competent care with evidence-based practices during birth in a supportive environment. Quality care includes efficient clinical and nonclinical interventions, a health staff with optimum competence, and a strong health infrastructure to obtain better health outcomes and ensure the positive experience of women and healthcare providers. Moreover, quality of care is considered a key component of the right to health and the route to equity and dignity for women and children. In order to achieve this, healthcare needs to be safe, effective, timely, efficient, equitable, and people-centered [12]. To achieve this, midwives/nurses/physicians should be trained to feel more confident with practices that facilitate normal childbirth, encouraged to more carefully assess the potential consequences and risks that come with each intervention, and to resort to interventions only when the situation calls for it, while mothers should also be educated to raise their awareness so that they can actively participate in the decision-making process with regard to how the child should be delivered. Women should be properly informed before any intervention is made and their approval should be sought [8, 10].

We believe that with an evidence-based approach to childbirth, useless treatment methods and unnecessary practices will be abandoned, women’s expectations will gain more weight leading to higher levels of maternal satisfaction, and costs will be reduced. The aim of this study is to examine the use of routine interventions in labor and maternal satisfaction at birth. In addition, the results of this study will contribute to the current literature on childbirth, filling a significant gap of knowledge regarding the frequency of routine interventions implemented during labor and the impact of such practices on maternal satisfaction.

## Methods

This descriptive study was conducted at the Turkish Ministry of Health, Kars Harakani Regional Training and Research Hospital over the period May 13–December 1, 2015. The study’s target population comprised all women who fulfilled the study criteria and who experienced spontaneous vaginal childbirth. The study sample included 351 women aged between 19 and 45 who gave birth normally and at term, had healthy fetuses, no chronic diseases, who did not experience complications during pregnancy, childbirth and the postpartum period, and who were admitted to the delivery ward in the latent stage of labor.

Using the sample selection formula for a known universe size, the sample size was calculated as an optimum 347 with a 95% confidence interval, a 5% margin of error, 5% significance, and 50% prevalence (due to the lack of prior knowledge). The study was completed with 351 participants. A year prior to the study, the number of women who gave birth by normal labor at the hospital was 3470.

Questionnaires, observation forms, and the Scale for Measuring Maternal Satisfaction in Vaginal Birth **(**SMMSVB) were used to collect the study data. After an examination of the related literature, some sociodemographic and obstetric characteristics of the women were included in the questionnaire, while the observation form contained a list of practices performed in the first, second, third and the early postpartum stages of labor [2–9, 11].

Two methods were used for collecting the study data. In the first stage, all interventions carried out during the period from the latent to the early postpartum stage, starting with the admission of the expecting mother to the delivery ward, were duly observed by the investigator and recorded in the observation form. The data collected through observations were corroborated by entries from records kept by the delivery staff. At this stage, all women who came to the delivery room in spontaneous labor were randomly selected using the simple random sampling technique and recorded as participants in the study by the researcher. In the second stage of data collection, an evaluation was made of the women’s satisfaction with the birth**.** The researcher administered the satisfaction scale to the participants immediately before their discharge (between the 20th–24th hours) via a face-to-face interview in the postpartum room.

Written and verbal informed consent was received from the pregnant women (regardless of their socio-demographic-obstetric characteristics)) admitted for delivery (in the delivery room in the first admissions stage) were provided with information about the study and informed that observations would be made at any time throughout the stages of the delivery. Because the relevant ethics research unit of the hospital demands both written and verbal consent for such studies (a descriptive, cross-sectional and observational study). Prior to the implementation of the data collection instruments and before the observations, the pregnant women were explained the purpose of the study in compliance with the principle of “Informed Consent,” their willful participation in the study was ensured in line with the principle of “Respect for Autonomy,” and they were assured that the information obtained about them would be kept confidential in accordance with the principle of “Confidentiality and the Protection of Privacy.” İnstitution approval (No: 82134845/730.08.03) was obtained from the the Kars Harakani Regional Training and Research Hospital, Turkey. İnstitution consent was preferred in our context without violating the ethical principles and it was approved by the committee.

*Scale for Measuring Maternal Satisfaction in Vaginal Birth* ***(*** *SMMSVB):*

Developed by Güngör and Beji and with its validity and reliability confirmed, this scale is a 5-point Likert-type instrument consisting of 43 items and 10 sub-dimensions. Thirteen items are reversely scored. The reversely scored items are converted first to calculate the scale score. The sum of the points of all the items on the scale yields the “total scale score” after the reversely scored items have been converted. The total raw score ranges from 43 to 215. A higher score on the scale indicates a higher level of maternal satisfaction in terms of the care received at the hospital for normal delivery. The cut-off score of the scale was 150.5 (≥150.5 = high maternal satisfaction level, < 150.5 = low maternal satisfaction level) [11].

### Data analysis

Data from the study were evaluated using the SPSS 21.0 statistical package program. Nonparametric tests were used in the data analysis as it was seen that the scale scores had no normal distribution (*p* < 0.05) when the scale’s normality distribution was examined (Kolmogorov - Smirnov). The Mann Whitney U test and other descriptive statistical methods [(frequency, percentage, median, interquartile range (IQR)] were also employed. The results were evaluated at a 95% confidence interval.

## Results

Of the surveyed women, 57.8% were between the ages 20–29, 40.7% had a maximum elementary level education, 90% were unemployed, 44.2% lived in rural areas, 69.5% were in the middle household income bracket, 84.3% had social security, 54.7% lived in extended families, 64.9% were multiparous, 87.5% had planned their pregnancies and 56.1% had received antenatal education (Table 1).

**Table 1:** Socio-demographic and obstetric characteristics of the women

Characteristics		Number	Percent
Age	15–19	39	11.1
	20–29	203	57.8
	30 and up	109	31.1
Education level	Primary school and below	143	40.7
	Secondary school	95	27.1
	High school	60	17.1
	College and university	53	15.1
Employment status	Yes	35	10.0
	No	316	90.0
Place of residence	Village	155	44.2
	District	73	20.8
	Province	123	35.0
Household income	Low	75	21.4
	Medium	244	69.5
	High	32	9.1
Health coverage	Yes	296	84.3
	No	55	15.7
Family structure	Extented	159	45.3
	Nuclear	192	54.7
Number of pregnancies	1	123	35.0
	2	92	26.2
	3	66	18.8
	4 and up	70	19.9
Unwilling pregnancy	Yes	307	87.5
	No	44	12.5
Receiving prenatal education	Yes	197	56.1
	No	154	43.9

The level of maternal satisfaction among the women in our study was 139.59 ± 29.02, which is low (cut-off scale score 150.5). The birth satisfaction of the women was evaluated according to the interventions they experienced during the three stages of birth (In **the first stage of labor:** perineal shaving, enema, induction, continuous EFM, palpation of contractions on the fundus, listening to fetal heartbeat via doppler/fetoscopy, movement restrictions, two-hourly vaginal examinations, amniotomy, analgesic medication for pain control, intravenous fluids, and nutrition/liquid intake restriction; **in the second stage of labor,** episiotomy, pushing techniques, fundal pressure, vacuum/forceps, vaginal irrigation with chlorhexidine, perineum protection using the “hands-on” method, early clamping of the umbilical cord, early skin-to-skin contact; and **in the third stage of labor,** removal of the placenta with controlled cord traction, bleeding control in the early postpartum period, uterine massage)**.**

In the first stage of labor, the women underwent the following procedures: 22.2%, perineal shaving; 80.6%, enema; 70.7%, oxytocin induction (elective: 67.3%); 95.4%, continuous EFM; 54.7%, palpation of contractions on the fundus; 92.3%, listening to fetal heartbeat with Doppler/fetoscopy; 35.0%, movement restrictions; 72.9%, two-hourly vaginal examinations; 31.9%, amniotomy; 31.3%, analgesic medication for pain controş; 74.9%, intravenous fluids; and 80.3%, nutrition/liquid intake restriction (Table 2).

**Table 2:** The distribution of the median scores obtained from SMMSVB according to the interventions at normal birth

Interventions in labor		Total scores of maternal satisfaction assessment scale at normal birth		
		N (%)	Median (IQR)	p*
In the first stage of labor				
Perineal shaving	Yes	78 (22.2)	127.5 (101.7–143)	0.158
	No	273 (77.8)	120 (100–150)	
Enema	Yes	283 (80.6)	123 (100–150)	0.607
	No	68 (19.4)	116 (105–150)	
Oxytocin induction	Yes	248 (70.7)	106 (94.2–138)	0.005
	No	103 (29.3)	116 (100–153)	
Continuous Electronıc Fetal Monıtorıng	Yes	335 (95.4)	117 (100–146)	0.021
	No	16 (4.6)	158.5 (123–183.7)	
Palpation of contractions	Yes	192 (54.7)	121 (88.2–142)	0.024
	No	159 (45.3)	146 (110–182)	
Fetal heart sound with Doppler /fetoscopy	Yes	324 (92.3)	108 (98–140)	0.021
	No	27 (7.7)	146 (105–157)	
Restricted movement	Yes	123 (35.0)	126 (88–156)	0.000
	No	228 (65.0)	134 (107–168.7)	
Vaginal examinations (two hourly)	Yes	256 (72.9)	122.5 (97–145.7)	0.001
	No	95 (27.1)	143 (102–181)	
Amniotomy	Yes	112 (31.9)	122.5 (98–158.7)	0.001
	No	239 (68.1)	132 (100–159)	
Administration of analgesics	Yes	110 (31.3)	138 (113.2–179.7)	0.007
	No	241 (68.7)	126 (100–151.5)	
Intravenous fluids	Yes	263 (74.9)	114 (98–143)	0.034
	No	88 (25.1)	132 (109–157)	
Restriction of liquids/nutrition	Yes	282 (63.0)	121.5 (100–145)	0.014
	No	69 (37.0)	145 (105.5–168)	
In the second stage of labor				
Chlorhexidine vaginal irrigation	Yes	344 (98.0)	126 (102–159)	0.877
	No	7 (2.0)	113 (95–143)	
Pushing techniques	“Open” Glottis	89 (25.4)	134 (107–157)	0.000
	“Closed” glottis	262 (74.6)	119 (100–144)	
Fundal pressure	Yes	152 (43.3)	121 (98–150)	0.007
	No	199 (56.7)	134 (108–157)	
Episiotomy	Yes	242 (68.9)	109 (99–145.2)	0.004
	No	109 (31.1)	136 (117–165)	
Manual protection of perineum	Hands off	51 (14.5)	126 (100–153)	0.519
	Hands on	300 (85.5)	122 (101–150)	
Vacuum / Forceps application	Yes	5 (1.4)	116 (95–135)	0.155
	No	346 (98.6)	126 (102–151)	
Time of umbilical cord clamping	Early	194 (55.3)	133 (108–168)	0.039
	Delay	157 (44.7)	114 (100.5–150)	
Skin-to-skin	Yes	49 (14.0)	147 (108.5–180)	0.000
	No	302 (86.0)	126 (102.7–152)	
In the third stage of labor				
Removal techniques of the placenta	With controlled cord traction	211 (60.1)	119 (100–150)	0.002
	Spontaneously	140 (39.9)	131 (106.2–164)	
Control of bleeding in early postpartum period	Yes	242 (68.9)	124 (101–153)	0.730
	No	109 (31.1)	126 (101–144)	
Uterine massage	Yes	97 (27.6)	117 (100.5–152)	0.243
	No	254 (72.4)	127 (102–150)	

*Mann Whitney U test

Accordingly, in the second stage of labor, the following procedures were carried out: 68.9%, episiotomy, 74.6%, pushing techniques (closed glottis); 43.3%, fundal pressure; 1.4%, vacuum/forceps; 99.1%, vaginal irrigation with chlorhexidine; 85.5%, perineum protection with the “hands-on” method; 55.3%; early clamping of the umbilical cord; and 86.0% did not engage in skin-to-skin contact in the early stage (Table 2).

In the third stage of labor**,** the placenta was removed with controlled cord traction in 60.1% of the women, 68.9% underwent bleeding control in the early postpartum period, and 27.6% were applied uterine massage (Table 2).

In the median (IQR) analysis, according to the cut-off score of the maternal satisfaction scale (cut-off scale score 150.5: ≥150.5 = high maternal satisfaction level, < 150.5 = low maternal satisfaction level), women who underwent the following interventions had lower scores of maternal satisfaction when compared to the women who did not: women who were induced (*p* = 0.005 < 0.05), who experienced continuous EFM (*p* = 0.021 < 0.05), palpation of contractions on the fundus (*p* = 0.024 < 0.05), whose fetus’ heartbeat was listened to through the abdomen (p = 0.021 < 0.05), women who experienced restricted mobility (*p* = 0.000 < 0.05), received two-hourly vaginal examinations (*p* = 0.001 < 0.05), were not administered analgesic medication (*p* = 0.007 < 0.05), were administered intravenous fluids infusion (*p* = 0.034 < 0.05), were subjected to restricted liquid/nutrition intake (*p* = 0.014 < 0.05), made to push using the closed glottis technique (*p* = 0.000 < 0.05), who underwent the application of fundal pressure (*p* = 0.007 < 0.05), had an episiotomy (*p* = 0.004 < 0.05), delayed umbilical cord clamping (*p* = 0.039 < 0.05), had the placenta removed with controlled cord traction (*p* = 0.002 < 0.05). Women who underwent amniotomy were found to have higher levels of satisfaction (*p* = 0.000 < 0.05) (Table 2).

## Discussion

*“First know what is normal. Expect what is normal and do not intervene when the state is normal! If a pathological condition develops, choose the correct intervention to bring the mother and baby back to normal state and apply it. Every intervention has a powerful impact and sometimes that impact may lead to the development of further pathologies, moving the situation further away from normality. Extra caution and care is advised when choosing which intervention to apply!” *[1, 13].

There have been important changes in the management of birth over the last 30 years. One trend is toward more natural childbirth, emphasizing the human emotional aspects of labor and delivery and seeing the mother as an active participant in the birth process rather than a baby-producing machine [2]. In addition to paying attention to the wellbeing of mother and child, an attempt is made to decrease unnecessary interventions at birth, protect mothers’ choices during the process, and reduce the cost of care [2, 4]. In Turkey, however, it has been reported that routine interventions, especially those used to accelerate the birth, are over-utilized [11, 14]**.** In the present study, two out of every three women were administered an enema, underwent elective induction, continuous EFM, had the fetus’ heart sounds listened to with Doppler/fetoscopy, experienced frequent vaginal examinations, had restrictions in intravenous fluids and nutrients and other intrapartum interventions in the first stage of labor. Almost one out of every three women experienced perineal shaving, palpation of contractions on the fundus**,** movement restrictions, amniotomy, and the administration of analgesics for pain control. These women however did not display satisfaction with these interventions. Similarly, 92.7% of the women in another study in Chile experienced medically augmented labor (artificial rupture of the membranes, continuous fetal monitoring, no oral hydration, while almost all received intravenous hydration, oxytocin, epidural analgesia, episiotomy, and most delivered in the lithotomy position). One-third of the women reported dissatisfaction with the care they received [4]. However, international organizations and evidence-based studies suggest that there is no need to restrict water and nutrient intake during labor at non-risk births [15, 16], no routine perineal shaving [17, 18], and enema should be applied at birth [19], delivery pain should be relieved [20, 21], intravenous fluids are not beneficial or harmful at birth [22, 23], women should be encouraged to take the position they are most comfortable during the birth, they should be allowed to move freely and their upright positions should be supported [24], vaginal examinations at 4-h intervals in the first stage of labor are adequate [25] and induction without indication and early amniotomy should not be applied as they would cause serious complications [26–29]. However in this study, **s**urprisingly, 31.9% of the women who participated in the study and received amniotomy were found to be satisfied with the practice. It is thought that women’s satisfaction with this practice has to do with midwives/nurses telling women they are performing amniotomy “to speed up the child’s delivery.”

In the present study, one reason for continuous EFM or frequent monitoring of the fetal heart beat with Doppler/fetoscopy may be tied to the fact that in Turkey gynecologists and obstetricians are the group, after general practitioners, that receive the greatest number of complaints of malpractice and therefore this group of specialists prefers to apply this practice to avoid complaints and paying costly compensation amounts [30]. Evidence-based studies, ACOG and NICE suggest that continuous EFM not be used in low-risk pregnancies and even that intermittent auscultation is a “convenient and safe alternative” [31–33]. In fact, palpation of contractions on the fundus is not a common practice that is used by midwives/nurses in normal birth management because of the widespread use of continuous EFM. However, those who are new to the profession and student midwives/nurses perform it to hone their skills and gain experience.

In this study, a large majority of the women in the 2nd stage of labor underwent an episiotomy, performed Valsalva pushing, received fundal pressure, vaginal irrigation with chlorhexidine, were offered perineal protection with a “hands on” technique, had the umbilical cord clamped early and were not exposed to skin-to-skin contact at an early stage. These interventions proved to have a negative impact on the women’s maternal satisfaction. However, evidence-based studies and available data do not provide any convincing evidence to support intrapartum vaginal irrigation with chlorhexidine to reduce the risk of maternal and neonatal infection [34] and the ideal clinical practice is to support spontaneous pushing and to encourage women to choose their own pushing techniques [35], intact perineal ratios are high and anal sphincter tears are frequently seen in women under fundal pressure [36], using limited episiotomy (mediolateral) when needed, reporting that routine episiotomy does not prevent pelvic floor injury [37–39], the perineum should not be touched during the second stage of birth until crowning [40], and umbilical cord be clamped not too soon (approximately at minutes 1–3, after the cord pulse stops) in terms of achieving positive neonatal outcomes, and that the maternal-infant relationship should be started as early as possible [41–43].

In this study, more than half of the women in the 3rd stage of labor had the placenta removed with controlled cord traction, bleeding control was achieved in the early postpartum period, and one-third were administered uterine massage. It was however found that women who experienced the removal of the placenta by controlled cord traction displayed a low level of maternal satisfaction. Evidence based studies, the routine performed of controlled umbilical cord traction by experienced health professionals using uterotonics, such as oxytocin [44, 45], and postpartum uterine massage performed every 10 min for a duration of 60 min reduced blood loss and the need for additional uterotonics, reducing the number of women experiencing more than 500 ml of blood loss by 50% [46, 47]. It is assumed that women are not satisfied with the practice because of the pain and sensitivity created by the pressure on the fundus during controlled cord traction.

Birth and maternal satisfaction in this period, which is seen as a very important experience in a woman’s life, is of the utmost importance in terms of the woman’s own health, the baby’s health and a positive family relationship [48]. Giving birth safely by receiving adequate and effective medical assistance is the principal expectation of a woman [47]. For this reason, unless there is a serious problem, most women do not want medical interventions that are performed to accelerate or facilitate the birth such as oxytocin, induction, enema, amniotomy, vacuum, fundal pressure, etc. [14]. It is therefore thought that interventions at birth affect childbirth satisfaction. However, findings in this study showed that despite recommendations provided by WHO in 1985 and further confirmed in 2015 by the most important related international associations, obstetric procedures are still over-utilized [12, 44]. Such interventions and restrictions cause women to have limited mobility, restricting their freedom of movement, making them feel less comfortable and experiencing more pain and anxiety because of not being able to direct their attention to other things other than lessening the impact of contractions. These procedures disrupt the hormonal balance of birth, protract the labor process, wear down the mother, cause the baby distress, increase the likelihood of an interventional birth, turn childbirth into a distressing experience, and reduce maternal satisfaction. The literature also supports the theory that obstetric intervention is linked with reduced birth satisfaction [6, 11, 14, 49]. Although maternal satisfaction is influenced by many factors, the prevailing view is that having a sense of control over the process, labor pain, personal support, expectations about childbirth, and medical interventions play a key role in maternal satisfaction [50]. It is especially having that sense of control over the birth process that determines maternal satisfaction levels. However, administering medications and excessive routine interventions cause women to lose all sense of control over the process, resulting in maternal dissatisfaction and postpartum complications [51]. The study conducted in accordance with the current literature found that the average satisfaction score for women who underwent routine interventions was 139.59 ± 29.02, which is low. That is to say, the women were not satisfied with induction, EFM, palpation of contractions on the fundus, movement restrictions, frequent vaginal examinations, lack of labor pain relief interventions, IV fluid infusions, fundal pressure, episiotomy, delayed cord clamping, removal of the placenta with controlled cord traction, and delayed skin-to-skin contact. Similarly, Binfa et al. [3] reported that although the majority of perceptions of wellbeing during labor was adequate or optimum, it is concerning that almost 1 out of every 4 mothers reported their general wellbeing as poor. It is remarkable that in Brazil, where unnecessary interventions are less applied, women have a higher and optimum level of birth satisfaction. The findings from this study are aligned with many of the categories of mistreatment identified in a systematic review of the global literature on mistreatment of women during labor and childbirth [52]. Chalmers and Dzakpasu [53] reported that among women having vaginal births, fewer interventions during labour was significantly associated with higher overall satisfaction with the labour and birth experience (ranging from 75% of women having no interventions to 46.4% having eight or more interventions rating their experiences as ‘very postive’). The WHO affirms that disrespectful treatment violates the rights of women and also infringes on their health, bodily integrity, right to life and freedom from discrimination. In this context, instead of traditional care services focusing on morbidity and mortality, efforts to provide women in participatory models of antenatal care are recommended to promote women-centered care in accordance with the WHO guidelines. This approach requires respect and familiarity for the childbearing woman and her family’s psychological, social, and cultural needs. Therefore, the focus and evaluation of care must be centered on emotional, social, and cultural aspects, rather than solely on the physical dimension [12, 33].

### Limitations

Several limitations of this study should be noted. First, the present findings are based on a cross-sectional survey. Second, the fact that the results are representative only for the institutions in a province of Turkey where the study was conducted was accepted as the limitation of the study. Third, iIn this study almost every woman was intervened at least once, so each intervention was compared with the general satisfaction status. Finally, further multivariate analyses are needed, and planned, to explore whether the observations emerging from this analysis of independent “Interventions during labor and maternal satisfaction” variables are robust or influenced by more complex associations in the data.

## Conclusion

Unnecessary interventions without medical indications spoil the physiology of birth. A birth where physiology is spoiled is traumatic for the mother, hazardous for the baby and exhausting for the physician/midwife/nurse. Our study results show that interventions not supported by evidence-based studies (such as continuous EFM, enema, induction, frequent vaginal examinations, food/liquid restrictions, the closed glottis pushing technique, episiotomy, movement restrictions, manual preservation of the perineum, early clamping of the umbilical cord, delayed skin-to-skin contact) were routinely performed at the discretion of the medical staff and that the women were not happy with this. The women were not properly informed about the procedures performed on them and their approval was not sought. Using clinically proven practices at all stages of labor instead of traditional practices and methods based on personal experiences will ensure standardization of the care provided and increase maternal satisfaction. Accordingly, health professionals should be encouraged to participate in on-the-job training programs and follow up on current trends in medical care. Additionally, qualitative aspects, such as the satisfaction of the woman and her family with the productive process, must also be assessed and the health professionals (midwife/nurse and physician) must also use appropriate precautions to ensure that interventions do not impose unnecessary risks for the women. Unintended consequences of intrapartum interventions make it imperative that educators cooperate with nurses, midwives, and physicians to promote natural processes for childbirth and advocate for policies that focus on ensuring informed consent and alternative options.

## Abbreviations

EFM: Electronic fetal monitoring
FHR: Fetal Heart Rate
FIGO: International Gynecology and Obstetrics Federation
HIV: Human Immunodeficiency Virus
ICM: International Midwives Confederation
ICSI: Institute for Clinical Systems Improvement
IV: Intravenous
NICE: National Institute of Health and Clinical Excellence / UK
RCM: The Royal Collage of Midwifes
SMMSVB: Scale for Measuring Maternal Satisfaction in Vaginal Birth
WHO: World Health Organization
